# ABO blood group as a determinant of COVID-19 and Long COVID: An observational, longitudinal, large study

**DOI:** 10.1371/journal.pone.0286769

**Published:** 2023-06-02

**Authors:** Joan B. Soriano, Adrián Peláez, Xavier Busquets, María Rodrigo-García, Elena Ávalos Pérez-Urría, Tamara Alonso, Rosa Girón, Claudia Valenzuela, Celeste Marcos, Elena García-Castillo, Julio Ancochea

**Affiliations:** 1 Servicio de Neumología, Hospital Universitario de la Princesa, Madrid, Spain; 2 Facultad de Medicina, Universidad Autónoma de Madrid, Madrid, Spain; 3 Centro de Investigación Biomédica en Red de Enfermedades Respiratorias (CIBERES), Instituto de Salud Carlos III, Madrid, Spain; 4 Laboratory of Molecular Cell Biomedicine, University of the Balearic Islands, Palma, Spain; Universidad Cientifica del Sur, PERU

## Abstract

**Background:**

An association of ABO blood group and COVID-19 remains controversial.

**Methods:**

Following STROBE guidance for observational research, we explored the distribution of ABO blood group in patients hospitalized for acute COVID-19 and in those with Long COVID. Contingency tables were made and risk factors were explored using crude and adjusted Mantle-Haentzel odds ratios (OR and 95% CI).

**Results:**

Up to September 2022, there were a total of 5,832 acute COVID-19 hospitalizations in our hospital, corresponding to 5,503 individual patients, of whom blood group determination was available for 1,513 (27.5%). Their distribution by ABO was: 653 (43.2%) group 0, 690 (45.6%) A, 113 (7.5%) B, and 57 (3.8%) AB, which corresponds to the expected frequencies in the general population. In parallel, of 676 patients with Long COVID, blood group determination was available for 135 (20.0%). Their distribution was: 60 (44.4%) from group 0, 61 (45.2%) A, 9 (6.7%) B, and 5 (3.7%) AB. The distribution of the ABO system of Long COVID patients did not show significant differences with respect to that of the total group (p ≥ 0.843). In a multivariate analysis adjusting for age, sex, ethnicity, and severity of acute COVID-19 infection, subgroups A, AB, and B were not significantly associated with developing Long COVID with an OR of 1.015 [0.669–1.541], 1.327 [0.490–3.594] and 0.965 [0.453–2.058], respectively. The effect of the Rh+ factor was also not significant 1,423 [0.772–2,622] regarding Long COVID.

**Conclusions:**

No association of any ABO blood subgroup with COVID-19 or developing Long COVID was identified.

## Introduction

The COVID-19 pandemic is far from over [1–3]. Now we know that many COVID-19 patients do not fully recover, and suffer for months (or years) a condition called Long COVID [4, 5]. This new yet ill-defined condition [6], encompasses a series of symptoms and ailments that has received many names [7]. It has been recently estimated that about one in seven individuals do not fully recover after the acute infection with SARS-CoV-2 at twelve months [8].

The available information about risk and protective factors of incident COVID-19 and Long COVID is rich and still growing [9]. In 2020, it was hypothesized that the ABO blood group could be related to the predisposition to and severity of COVID-19 [10]. Contrary to other determinants, this relationship of ABO blood group with COVID-19 has been highly controversial. A number of systematic reviews and meta-analyses are already available, signaling to a modest association, initially with type A and later with AB [11–14]. Notably, others have meta-analysed the same evidence, yet with inconclusive findings [15].

It is established that differences in ABO blood group antigen expression can increase or decrease host susceptibility to a number of infectious agents [16], including viruses [17–20]. It is also well established that SARS-CoV-2 binds to human cells through the angiotensin-converting enzyme II (ACE-2) receptor. Red blood cells precursors express ACE-2 receptor and CD147 at day 5 of differentiation, which makes them susceptible to SARS-CoV-2 infection [21].

However, any association of ABO blood types with COVID-19 remains questionable. Finally, to date a possible association of the ABO group with the development of Long COVID has not been explored, except from a small study from Najaf, Iraq [22].

Our objective is to determine the association of ABO group types (if any) in hospitalized patients admitted acutely for COVID-19, and to explore it also in patients with Long COVID.

## Methods

This is an observational, longitudinal study in a prospective cohort of hospitalized patients with COVID-19 admitted at the Hospital de La Princesa, Madrid, Spain from February 2020 to September 2022. We obtained information from clinical records of all adult patients (age ≥ 18 years) with a positive COVID-19 clinical diagnosis upon hospital admission, confirmed either by positive antigen or polymerase chain reaction (PCR) tests. Further details about this cohort are available elsewhere [23, 24]. The research protocol was approved (CEIm 10/21 with N° 4.468). No individual consent form was required, as we only use and report unidentified administrative data.

Briefly, we were initially interested on determinants and clinical outcomes from any COVID-19-related admission up to 30 days post discharge, which was obtained from the electronic medical records of our hospital system. The biometric, laboratory (blood and urine), and comorbidity variables were obtained and analyzed following STROBE guidelines for observational research [25]. ABO blood group was determined in the cohort of patients hospitalized for acute COVID-19 and in those patients with Long COVID seen in the post-COVID consultation. For reference population, we used published statistics [26, 27].

### Statistical methods

A first descriptive analysis of the patients’ characteristics was performed by calculating central tendency and dispersion measures of quantitative variables. For qualitative variables, comparison of proportions was tested by using the χ^2^ test or the Fisher exact test, whenever necessary. Contingency tables were made and risk factors were explored using crude and adjusted Mantle-Haentzel odds ratios (OR and 95% CI). In addition to descriptive statistics, a Kaplan-Meier analysis of in-hospital mortality up to 30 days post-discharge was performed. To obtain the hazards ratios, a Cox proportional regression was fitted over the statistically significant variables obtained from the bivariate analysis. A *p*-value below 0.05 was considered to indicate statistical significance in all analyses. Data management, statistical calculations, and graphical plots were conducted using the R statistical software.

## Results

**Fig 1:**
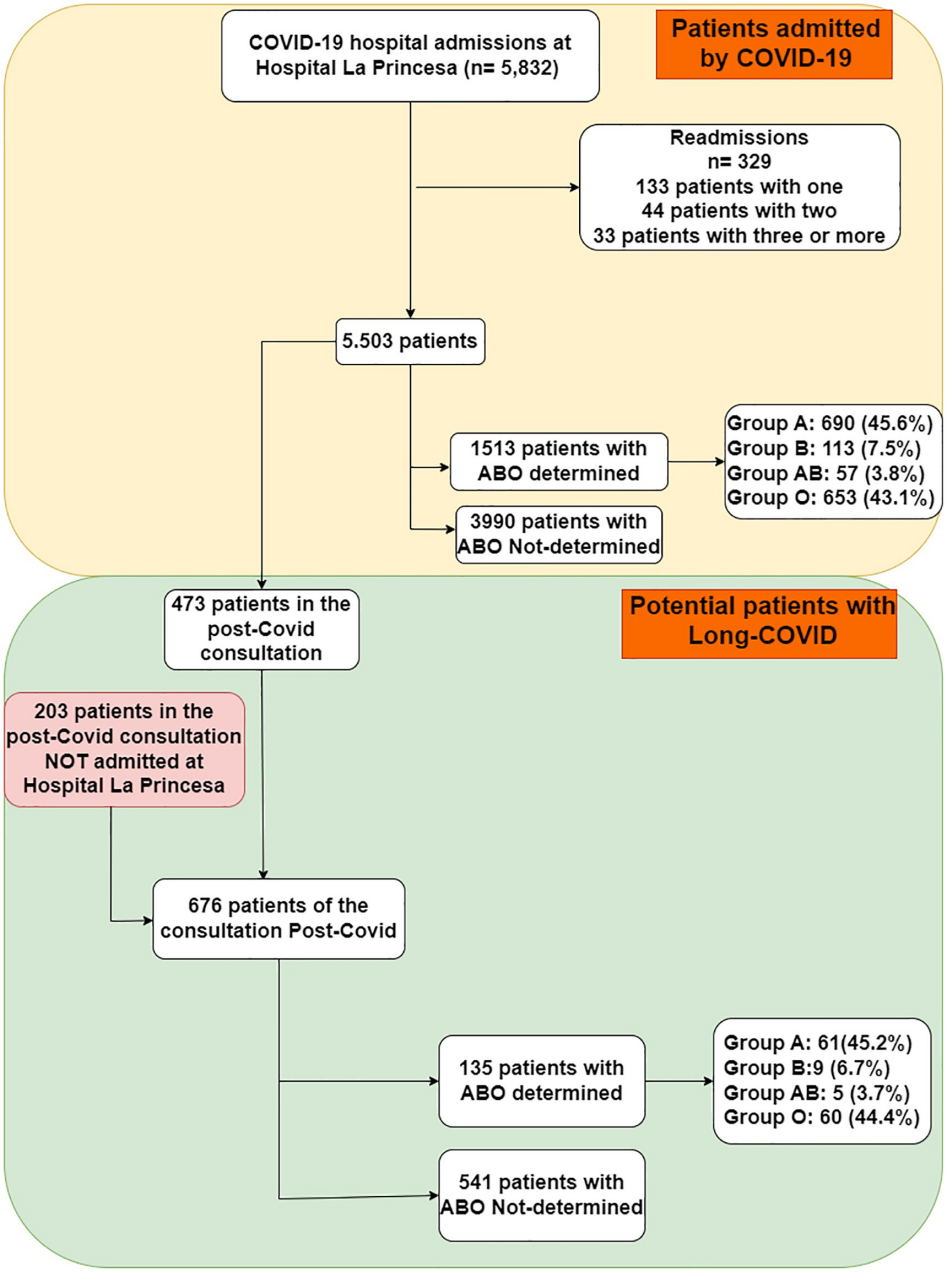
STROBE flowchart of participation in the study.

From February 2020 to September 2022, there were a total of 5,832 acute COVID-19 hospitalizations in our hospital, corresponding to 5,503 individual patients, of whom blood group determination was available for 1,513 (27.5%) (Fig 1). Of interest, those who had blood group assessed, were not significantly different from those not tested in their gender and smoking status distribution (S1 Table). However, they were on average, older, required a longer hospitalization and had worse clinical outcomes in all assessments (all p <0.05), likely related with the medical decision to type their blood group. Further, there were more persons of Latin-American origin (p<0.05), confirming our earlier publication (Table 1) [24].

**Table 1:** Demographic and clinical characteristics of patients admitted for COVID-19, and of the subsample with Long COVID.

	Patients admitted with COVID-19 (n = 1,513)	Patients with Long COVID (n = 135)	*p*-value
Age in years, m±SD	75.9±15.2	68.2±13.0	<0.001
Female, n (%)	712 (47.1%)	41 (30.4%)	<0.001
Latino ethnicity, n (%)	129 (8.5%)	26 (19.3%)	<0.001
Smokers, n (%)			
Former	310 (20.5%)	35 (25.9%)	0.137
Current	101 (6.7%)	8 (5.9%)	0.737
Duration of admission, m±SD	21.3±27.3	38.3±33.9	<0.001
Use of health services, n (%)	417 (27.6%)	78 (57.8%)	<0.001
NIMV	178 (11.8%)	49 (36.3%)	<0.001
IMV	165 (10.9%)	39 (28.9%)	<0.001
IRCU	87 (5.8%)	6 (4.4%)	0.529
ICU	293 (19.4%)	63 (46.7%)	<0.001
Death, n (%)	326 (21.5%)	0 (0.0%)	<0.001
Group ABO, n (%)			
O	653 (43.2%)	60 (44.4%)	0.773
A	690 (45.6%)	61 (45.2%)	0.925
B	113 (7.5%)	9 (6.7%)	0.733
AB	57 (3.8%)	5 (3.7%)	0.970
Group Rh, n (%)			
-	235 (15.5%)	17 (12.6%)	0.363
+	1278 (84.5%)	118 (87.4%)	0.363

Non-invasive mechanical ventilation (NIMV); Invasive mechanical ventilation (IMV); IRCU (Intermediate respiratory care unit); Intensive care unit (ICU).

In parallel, out of 676 patients with Long COVID seen in our post-COVID external consultation, blood group determination was available for 135 (20.0%) (Fig 1). In this subsample with Long COVID, those who had blood group assessed, were not significantly different from those not tested in their gender and smoking status distribution again (S2 Table), and in this case on Latino origin too; however, they were on average, older, required a longer hospitalization and had many (but not all) poorer adverse clinical outcomes (p <0.05).

**Fig 2:**
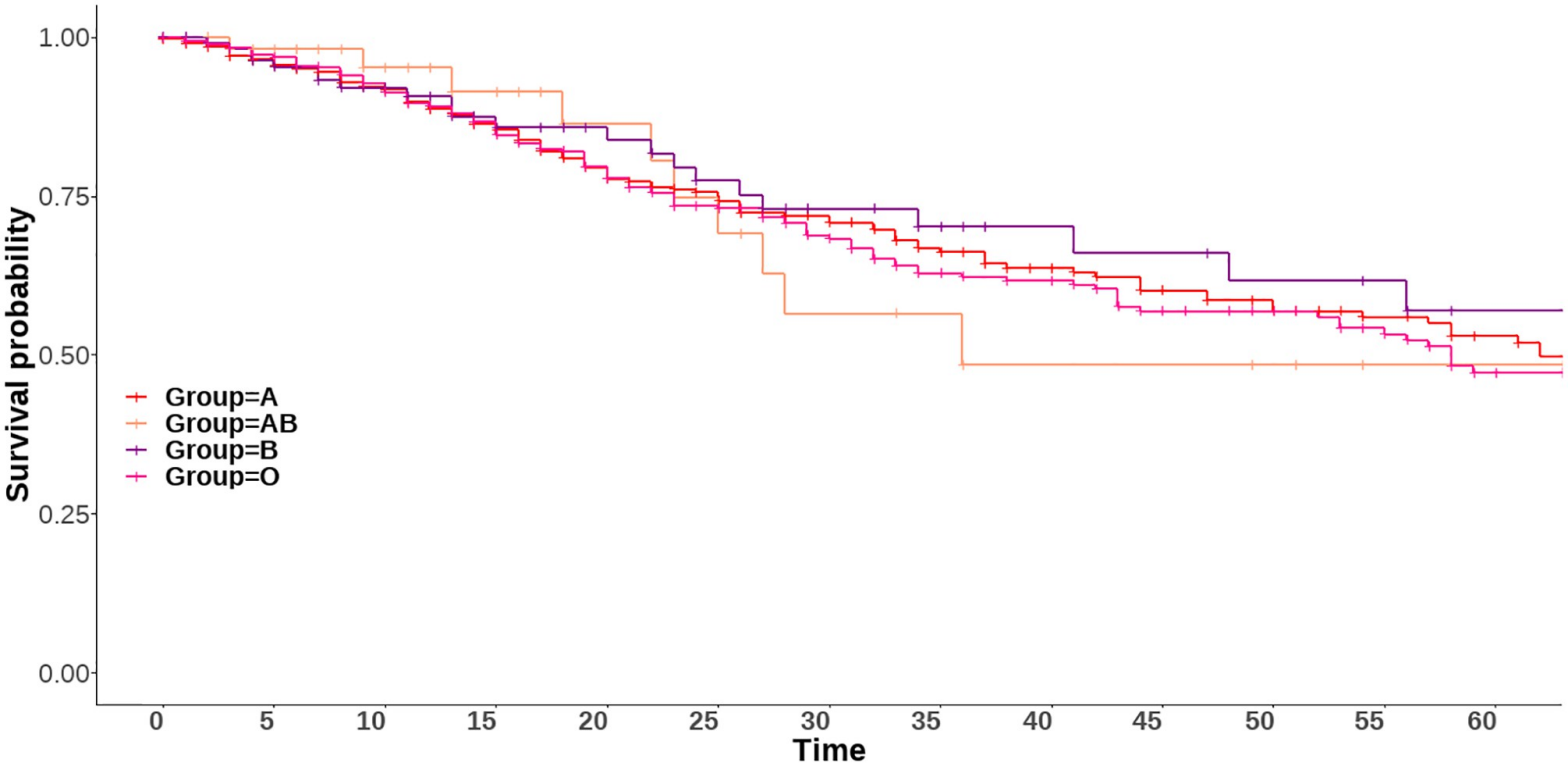
Kaplan-Meier in-hospital survival curves of patients admitted for COVID-19 by ABO blood group.

Among admitted patients with COVID-19, their distribution by ABO was: 653 (43.2%) group 0, 690 (45.6%) A, 113 (7.5%) B, and 57 (3.8%) AB (Table 1), which corresponds to the expected frequencies in the general Spanish population (See Methods as 45% belong to group 0, 42% to A, 10% to B, and 3% to AB) (p >0.05). Applying customary survival statistics in here, the Kaplan-Meier survival curves of patients admitted for COVID-19 up to 60-days post discharge were not statistically significant by all four ABO blood group types (Fig 2).

When exploring those with Long COVID, their ABO type distribution was: 60 (44.4%) from group 0, 61 (45.2%) A, 9 (6.7%) B, and 5 (3.7%) AB. And, the distribution of the ABO system of Long COVID patients did not show significant differences with respect to that of the total group of COVID-19 patients (p ≥ 0.843) (Table 1).

Finally, in a multivariate analysis adjusting for age, sex, ethnicity, and severity of acute COVID-19 infection, subgroups A, AB, and B were not significantly associated with developing Long COVID with an OR of 1.015 [0.669–1.541], 1.327 [0.490–3.594] and 0.965 [0.453–2.058], respectively. The effect of the Rh+ factor was also not significant 1,423 [0.772–2,622] regarding Long COVID (Table 2).

**Table 2:** Determinants of Long COVID (crude and adjusted OR with 95% C.I.).

Variable	Category	Crude Odds Ratio		Adjusted Odds Ratio	
		(95% C.I.)	*p*-value	(95% C.I.)	*p* value
**Age (years)**	18–29	Ref.	—	—	—
	30–39	1.909 (0.161–22.656)	0.608	1.944 (0.161–23.546)	0.601
	40–49	5.559 (0.656–47.077)	0.116	6.133 (0.71–52.948)	0.099
	50–59	3.758 (0.474–29.823)	0.210	4.58 (0.566–37.039)	0.154
	60–69	2.833 (0.367–21.854)	0.318	3.498 (0.444–27.574)	0.235
	70–79	2.682 (0.351–20.469)	0.341	3.460 (0.440–27.175)	0.238
	80–89	0.931 (0.119–7.311)	0.946	1.316 (0.162–10.688)	0.797
	90 and older	0.226 (0.022–2.266)	0.206	0.360 (0.035–3.736)	0.392
**Sex**	Male	Ref.	—	—	—
	Female	0.558 (0.377–0.826)	**0.004**	0.624 (0.412–0.946)	**0.026**
**Latino ethnicity**	Non-Latino	Ref.			
	Latino	3.464 (2.147–5.590)	**< 0.001**	2.33 (1.364–3.981)	**0.002**
**Smoker**	Non-smoker	Ref.	—	—	—
	Former-	1.694 (1.112–2.581)	0.014	1.424 (0.908–2.233)	0.124
	Current-	1.145 (0.536–2.445)	0.726	0.717 (0.328–1.568)	0.404
**ABO**	O	Ref.	—	—	—
	A	0.904 (0.608–1.344)	0.619	1.015 (0.669–1.541)	0.943
	B	0.960 (0.460–2.003)	0.913	0.965 (0.453–2.058)	0.927
	AB	1.067 (0.409–2.783)	0.895	1.327 (0.490–3.594)	0.577
**RH**	RH-	Ref.	—	—	—
	RH+	1.560 (0.862–2.824)	0.142	1.423 (0.772–2.622)	0.259

Risk of Long COVID among those admitted for acute COVID-19.

## Discussion

Beyond established risk factors of COVID-19 and of Long COVID, the search for novel determinants of both remains an unmet need [2, 28]. Our study confirms established risk factors for susceptibility to both conditions, such as middle age, Latin-American origin and former smoking status. However, we were unable to identify within our study population any relationship of ABO blood group types neither with acute COVID-19 nor with Long COVID.

Our research has some strengths, including: a large size; research spanning seven different pandemic waves lasting two full years; blinding on the primary aim of this research; and the multivariate statistics applied. However, a number of limitations must be highlighted. ABO blood group was only determined according to patient’s medical needs. As in S1 Table, those patients admitted with COVID-19 whose ABO blood group was determined were significantly older and had more severe disease overall. However, a potential type II error favors our negative conclusion. Other limitations include: inability to test patients who died quickly due to critical illness; single ethnic group, although those of LatinAmerican origin were further explored. Situation is likely very complex, influenced by successive vaccinations and boosters, different mechanism of Long COVID, and other factors [29].

### Plausibility and mechanisms

The mechanism of action underlying the association of blood type and viral infections is still ill known [30, 31]. Hypotheses are based on the fact that blood group antigens can function as protein receptors for viruses and as a consequence activate the endophagic cellular mechanisms that facilitate the intracellular uptake of the viruses [16]. The receptor-binding domain (RBD) of S protein of SARS-CoV-2 [32], located in the N-terminal part of the protein mediates ACE-2 binding [33], and this RBD is highly sensitive to neutralizing antibodies [34].

Guillon P, *et al*. [35], demonstrated in a cell culture-based binding assay mimicking the interaction between the S protein and ACE-2 [36], that natural antibodies of the ABO system block the S protein ACE-2 binding in SARS-CoV [37]. Natural anti-A or -B antibodies from group 0, B, and A individuals could bind to the S protein and impair its binding with ACE-2, preventing infection [11–14].

### Implications for the future

To date most studies have been small (low double-digit) [38], from a handful of countries [11–14], and not following STROBE guidance in full [25]. Therefore Type I error cannot yet be ruled out, so more evidence meta-analyzed from patients in all COVID-19 severities including those pauci-asymptomatic [39], and other geographical locations, is needed [40].

Within this research, comorbidities were not identified as independent drivers in our null association with ABO blood group. However, in our previous research comorbidities were found to be major drivers of health outcomes in COVID-9 and Long COVID, and we identified that both number of and specific comorbidities, such as obesity, previous chronic respiratory disease or cardiovascular disease [23, 24].

The situation might be different, and more complex, in Long COVID. Given there are different pathophyisiological mechanisms identified, it must be ruled out whether some/any mechanisms be associated with susceptibility/protection to develop it [41, 42]. We have to concur with Kim Y, et al. there does not appear to be any relationship between blood type and COVID-19–related severity of illness or mortality [15].

We conclude that in our study population there was no association identified of any ABO blood type with either suffering acute hospitalization for COVID-19 or later developing Long COVID.

## Supporting information

S1 Table STROBE non-response table comparing demographic and clinical characteristics of patients admitted for COVID-19 with/without ABO blood group determined. (DOCX)

S2 Table STROBE non-response table comparing demographic and clinical characteristics of patients with Long COVID with/without ABO blood group determined. (DOCX)

S1 File ICMJE disclosure form. (DOCX)

S1 Checklist STROBE statement—Checklist of items that should be included in reports of observational studies. (DOC)
