# Supplementary material for: ABO blood group as a determinant of COVID-19 and Long COVID: An observational, longitudinal, large study
Source: PLoS One. 2023 Jun 2;18(6):e0286769. doi: 10.1371/journal.pone.0286769 (PMC10237493; doi:10.1371/journal.pone.0286769)
Supplement: S1 Table — (DOCX) [file pone.0286769.s001.docx]

**S1 Table**. STROBE non-response table comparing demographic and clinical characteristics of patients admitted for COVID-19 with/without ABO blood group determined

|  | **Total**  ***n* =5,503** | **ABO**  **Not- determined**  ***n*=3,990 (72.5%)** | **ABO**  **Determined**  ***n*=1,513 (27.5%)** | ***P* value** |
| --- | --- | --- | --- | --- |
| Age in years, m±SD | 71.2 (±17.4) | 69.4 (±17.8) | 75.9 (±15.2) | <0.001 |
| Female, n (%) | 2598 (47.2%) | 1886 (47.3%) | 712 (47.1%) | 0.890 |
| Latino ethnicity, n (%) | 831 (15.1%) | 702 (17.6%) | 129 (8.5%) | <0.001 |
| Smokers, n (%) |  |  |  |  |
| Former | 1,120 (20.4%) | 810 (20.3%) | 310 (20.5%) | 0.877 |
| Current | 321 (5.8%) | 220 (5.5%) | 101 (6.7%) | 0.101 |
| Duration of admission, m±SD | 12.6 (±17.2) | 9.25 (±9.3) | 21.3 (±27.3) | <0.001 |
| Use of health services, n (%) | 860 (15.6%) | 443 (11.1%) | 417 (27.6%) | <0.001 |
| NIMV | 455 (8.3%) | 277 (6.9%) | 178 (11.8%) | <0.001 |
| IMV | 268 (4.9%) | 103 (2.6%) | 165 (10.9%) | <0.001 |
| IRCU | 113 (2.1%) | 26 (0.7%) | 87 (5.8%) | <0.001 |
| ICU | 511 (9.3%) | 218 (5.5%) | 293 (19.4%) | <0.001 |
| Death, n (%) | 800 (14.5%) | 474 (11.9%) | 326 (21.5%) | <0.001 |

**Table footnote**: Non-invasive mechanical ventilation (NIMV); Invasive mechanical ventilation (IMV); IRCU (Intermediate respiratory care unit); Intensive care unit (ICU)
