# Supplementary material for: ABO blood group as a determinant of COVID-19 and Long COVID: An observational, longitudinal, large study
Source: PLoS One. 2023 Jun 2;18(6):e0286769. doi: 10.1371/journal.pone.0286769 (PMC10237493; doi:10.1371/journal.pone.0286769)
Supplement: S2 Table — (DOCX) [file pone.0286769.s002.docx]

**S2 Table**. STROBE non-response table comparing demographic and clinical characteristics of patients with Long COVID with/without ABO blood group determined

|  | **Total**  *n* =676 | **ABO**  **Not- determined**  *n*=541 (80.0%) | **ABO**  **Determined**  *n*=135 (20.0%) | ***p-*value** |
| --- | --- | --- | --- | --- |
| missing, n (%) | 203 (30.0%) | 188 (34.8%) | 15 (11.1%) |  |
| Age in years, m±SD | 65.3 (±13.7) | 64.3 (±13.9) | 68.2 (±13.0) | 0.004 |
| Female, n (%) | 186 (27.5%) | 145 (26.8%) | 41 (30.4%) | 0.406 |
| Latino ethnicity, n (%) | 110 (16.3%) | 84 (15.5%) | 26 (19.3%) | 0.293 |
| Smokers, n (%) |  |  |  |  |
| Former | 121 (17.9%) | 86 (15.9%) | 35 (25.9%) | 0.007 |
| Current | 28 (4.1%) | 20 (3.7%) | 8 (5.9%) | 0.245 |
| Duration of admission, m±SD | 21.7 (±22.6) | 16.1 (±13.2) | 38.3 (±33.9) | <0.001 |
| Use of health services, n (%) | 229 (33.9%) | 151 (27.9%) | 78 (57.8%) | <0.001 |
| NIMV | 163 (24.1%) | 114 (21.1%) | 49 (36.3%) | <0.001 |
| IMV | 80 (11.8%) | 41 (7.6%) | 39 (28.9%) | <0.001 |
| IRCU | 14 (2.1%) | 8 (1.5%) | 6 (4.4%) | 0.042 |
| ICU | 136 (20.1%) | 73 (13.5%) | 63 (46.7%) | <0.001 |
| Death, n (%) | 0.0 (0.0%) | (0.0%) | (0.0%) | 1.000 |

**Table footnote**: Non-invasive mechanical ventilation (NIMV); Invasive mechanical ventilation (IMV); IRCU (Intermediate respiratory care unit); Intensive care unit (ICU)
